# Association between serum hepcidin-25 levels and hyporesponsiveness to erythropoiesis-stimulating agents in Japanese patients receiving hemodialysis: a cross-sectional study

**DOI:** 10.1007/s10157-025-02783-9

**Published:** 2025-10-31

**Authors:** Ryo Fujikawa, Nobuo Nagano, Yuko Mitobe, Kyoko Ito

**Affiliations:** 1 Medical Affairs Department, Torii Pharmaceutical Co., Ltd., 3-4-1 Nihonbashi-Honcho, Chuo-Ku, Tokyo, 103-8439 Japan; 2 Kidney Disease and Dialysis Center, Hidaka Hospital, Hidaka-Kai, 886 Nakao-Machi, Takasaki, Gunma 370-0001 Japan

**Keywords:** Hepcidin-25, Hyporesponsiveness to erythropoiesis-stimulating agents, Hemodialysis patients, Anemia in chronic kidney disease, Latex immunoassay

## Abstract

**Background:**

Hepcidin-25 plays an important role in regulating iron metabolism; however, the association between hepcidin-25 levels and hyporesponsiveness to erythropoiesis-stimulating agents (ESAs) is controversial. We aimed to clarify the associations between serum hepcidin-25 levels and hyporesponsiveness to ESAs in Japanese patients receiving hemodialysis, and between hepcidin-25 levels and other factors.

**Methods:**

This observational cross-sectional study included hemodialysis patients recruited at Heisei-Hidaka Clinic in Japan from August 2023 to June 2024. Serum hepcidin-25 levels were measured by latex immunoassay. Hyporesponsiveness to ESAs was determined by the ESA resistance index (ERI). The correlation between hepcidin-25 levels and ERI was evaluated using Pearson’s correlation coefficient. We also investigated the patient characteristics associated with hepcidin-25 levels using multiple regression analysis.

**Results:**

Hepcidin-25 levels were significantly negatively correlated with ERI (*r* = − 0.438, *p* = 0.0005). Hepcidin-25 levels also showed significant positive correlations with serum iron, transferrin saturation (TSAT), serum ferritin, and high sensitive C-reactive protein (hs-CRP), and significant negative correlations with hematocrit, unsaturated iron-binding capacity, total iron-binding capacity, and serum erythropoietin levels. Hepcidin-25 levels were significantly higher in the patients who received oral iron-containing preparations than in those without these preparations. Multiple regression analysis showed significant partial regression coefficients for ERI, hematocrit, TSAT, serum ferritin, hs-CRP, and the administration of oral iron-containing preparations.

**Conclusion:**

Serum hepcidin-25 levels were significantly negatively correlated with the ERI. The results suggest that hepcidin-25 levels might be associated with ERI, hematocrit, TSAT, serum ferritin, hs-CRP, and the administration of oral iron-containing preparations.

## Introduction

Anemia in chronic kidney disease (CKD) is caused by several factors, including reduced production of erythropoietin (EPO) and disrupted iron homeostasis due to renal dysfunction [1]. Hyporesponsiveness to erythropoiesis-stimulating agents (ESAs) has been observed in patients with iron deficiency and inflammation [2, 3], and has been associated with a poor prognosis in hemodialysis (HD) patients [4]. It is therefore important to eliminate the causes of ESA hyporesponsiveness.

Hypoxia-inducible factor-prolyl hydroxylase inhibitors (HIF-PHIs) have a newly described mechanism and have recently become available to treat anemia in CKD. HIF-PHIs produce endogenous EPO, and also improve the efficiency of iron utilization [5], including by down-regulating hepcidin levels. Hepcidin is an important peptide hormone related to iron metabolism, which is produced in the liver and regulates ferroportin, as a membrane protein that transports iron. Hepcidin-25 is a bioactive form of hepcidin [6]. Hepcidin-25 synthesis in the liver is elevated in patients with CKD, mediated by the inflammatory cytokine interleukin-6 (IL-6). In addition, the clearance of hepcidin-25 is decreased in these patients, resulting in increased serum levels [7], which in turn limit iron utilization and cause ESA hyporesponsiveness [8]. Although hepcidin-25 has been proposed as a key factor in anemia management in patients with CKD, the association between hepcidin-25 levels and ESA responsiveness remains controversial [9–12]. Clarifying this association in the Japanese context may thus help to guide the selection and monitoring of anemia therapies, including the use of HIF-PHIs. A new latex immunoassay (LIA) system using a specific antibody has recently been developed that allows the rapid measurement of hepcidin-25 levels [13]. Hepcidin-25 levels vary depending on the assay method [14], and the LIA results have shown a good correlation with results from conventional liquid chromatography/tandem mass spectrometry methods [13].

This study aimed to evaluate the association between serum hepcidin-25 levels measured by LIA and hyporesponsiveness to ESAs in Japanese patients undergoing HD, and to explore the associations between hepcidin-25 levels and other clinical and laboratory factors. Notably, hepcidin-25 was measured using a LIA method, which could be useful for routine laboratory testing.

## Materials and methods

### Study design and participants

This observational cross-sectional study was conducted at Heisei-Hidaka Clinic (Takasaki, Gunma, Japan) from August 2023 to June 2024. The study was registered in the Japan Registry of Clinical Trials (jRCT1031230345).

The inclusion criteria were: (1) Japanese patients aged ≥ 18 years at informed consent; (2) patients receiving HD [including hemodiafiltration (HDF)] three times a week for ≥ 12 weeks prior to informed consent; (3) patients who had been receiving ESA therapy for ≥ 8 weeks prior to informed consent with an interval of ESA administration ≤ 4 weeks; (4) patients who had been receiving the same ESA for 8 weeks prior to informed consent with the last two administration intervals and doses being the same; and (5) patients considered to have no health problems preventing participation in this study, as assessed by the principal investigators or co-investigators.

The exclusion criteria were: (1) patients with severe complications, such as cerebral, hepatic, renal, cardiac, pulmonary, gastrointestinal, hematological, endocrine, metabolic or psychiatric diseases; (2) patients who participated in other studies involving medical devices or interventional clinical studies within 12 weeks prior to the start of this study; and (3) patients assessed as unsuitable for inclusion by the principal investigators or co-investigators.

We enrolled 60 patients receiving low-dose (< 30 µg/week, *n* = 21), medium-dose (≥ 30 and < 60 µg/week, *n* = 20), or high-dose ESAs (≥ 60 µg/week, *n* = 19) of darbepoetin alfa. No patients were receiving HIF-PHIs.

### Data collection

After obtaining consent, we collected information on patient background, dialysis conditions, and laboratory measurements from the patients’ medical records. We also measured hepcidin-25, high sensitive C-reactive protein (hs-CRP), IL-6, and serum EPO levels that were not included in routine clinical practice. Hepcidin-25 and hs-CRP levels were measured by FUJIFILM Wako Pure Chemical Corporation (Osaka, Japan), while other laboratory measurements were provided by BML, Inc. (Tokyo, Japan). The laboratory measurements were analyzed for 60 patients, except for IL-6 and serum EPO levels, which were analyzed for 58 patients because of missing data. Blood was drawn immediately after the start of dialysis and before the administration of ESAs and intravenous iron preparations, to avoid any influence on hepcidin-25 levels. Serum hepcidin-25 levels were measured by LIA using a hepcidin-25 measuring reagent (FUJIFILM Wako Pure Chemical Corporation) and automated biochemistry analyzers (JCA-BM6050 and JCA-BM9130, JEOL Ltd., Tokyo, Japan; and TBA-c16000, Canon Medical Systems Corporation, Tochigi, Japan). The assay results provided hepcidin-25 levels from 0.1 ng/mL.

### Endpoints

The primary endpoint was the correlation between hepcidin-25 levels and the ESA resistance index (ERI). The ERI was calculated using a single time point as follows: dose of ESA administered (dose/week)/[dry weight (kg) × hemoglobin level (g/dL)]. The secondary endpoints were the correlations of hepcidin-25 levels with patient background, dialysis condition, and laboratory measurement parameters. Differences in hepcidin-25 levels among categories were evaluated for each parameter. Iron repletion was divided into four categories: Group I, ferritin ≥ 100 ng/mL and transferrin saturation (TSAT) ≥ 20%; Group II, ferritin < 100 ng/mL and TSAT ≥ 20%; Group III, ferritin ≥ 100 ng/mL and TSAT < 20%; and Group IV, ferritin < 100 ng/mL and TSAT < 20%. Independent factors affecting hepcidin-25 levels were analyzed by multiple regression analysis.

### Statistical analysis

The target sample size of 60 patients was determined based on previous reports [10]. Numeric data were expressed as mean ± standard deviation or median (interquartile range). Continuous variables that were not normally distributed were transformed into natural logarithms. Nominal variables were described as number of cases (percentage). The relationships of hepcidin-25 levels with ERIs and continuous patient background variables, including ESA dose, dialysis conditions, and laboratory measurement parameters were analyzed using Pearson’s product-moment correlation coefficients. For nominal variables, differences in hepcidin-25 levels in relation to patient background parameters and iron repletion were analyzed by Mann–Whitney U tests for comparing two categories, and the Steel–Dwass method for comparing three or more categories. Multiple regression analysis was performed with hepcidin-25 levels as an objective variable. No analyses were performed based on the three ESA dose categories of patients’ enrollment. All analyses were performed using SAS version 9.4 (SAS Institute Inc., Cary, NC, USA). Microsoft Excel 2016 (Microsoft, Redmond, WA, USA) was used for scatter plot. A *p*-value < 0.05 was considered statistically significant.

## Results

### Baseline characteristics of study participants

Sixty patients were enrolled in this study, and all patients received darbepoetin alfa. The baseline characteristics of the patients are shown in Table 1. The median hepcidin-25 level was 8.15 (1.00–23.85) ng/mL, the median ERI was 0.042 (0.023–0.086) µg/kg/g/dL. No adverse events were associated with the execution of this study.

**Table 1:** Baseline characteristics

Characteristic	*n* = 60
**Patient background**	
Sex (male), *n* (%)	41 (68.3)
Age (years), mean (SD)	68.3 (10.1)
Dry weight (kg), mean (SD)	61.42 (13.11)
BMI (kg/m^2^), mean (SD)	23.08 (4.28)
Primary disease of CKD, *n* (%)	
Chronic glomerulonephritis	18 (30.0)
Diabetic nephropathy	22 (36.7)
Nephrosclerosis	10 (16.7)
Others/Unknown	10 (16.7)
Comorbidity, *n* (%)	
Hypertension	55 (91.7)
Secondary hyperparathyroidism	49 (81.7)
Hyperphosphatemia	45 (75.0)
Diabetes	32 (53.3)
GNRI, mean (SD)	94.1 (6.5)
ERI (µg/kg/g/dL), median (IQR)	0.042 (0.023–0.086)
Intravenous iron, *n* (%)	12 (20.0)
Oral iron-containing preparations, *n* (%)	12 (20.0)
**Dialysis condition**	
Modality, *n* (%)	
Hemodialysis	4 (6.7)
Hemodiafiltration	56 (93.3)
Dialysis vintage (months), median (IQR)	66.0 (26.5–127.5)
Kt/V, mean (SD)	1.41 (0.29)
Dialysis time (minutes), mean (SD)	243.5 (12.5)
**Laboratory measurement**	
Hemoglobin (g/dL), mean (SD)	11.09 (0.96)
Hematocrit (%), mean (SD)	35.67 (3.02)
RBC (× 10^4^/µL), mean (SD)	371.0 (44.6)
MCV (fL), mean (SD)	96.7 (6.7)
MCH (pg), mean (SD)	30.10 (2.60)
MCHC (%), mean (SD)	31.09 (1.07)
Platelet (× 10^4^/µL), mean (SD)	19.85 (7.07)
Total bilirubin (mg/dL), median (IQR)	0.30 (0.20–0.40)
AST (U/L), median (IQR)	12.0 (9.5–15.0)
ALT (U/L), median (IQR)	9.5 (8.0–12.0)
γ-GTP (U/L), median (IQR)	19.0 (13.5–34.0)
Cholinesterase (U/L), mean (SD)	217.4 (47.7)
Hepcidin-25 (ng/mL), median (IQR)	8.15 (1.00–23.85)
Ferritin (ng/mL), median (IQR)	44.55 (30.40–71.45)
Serum iron (µg/dL), mean (SD)	56.8 (22.1)
UIBC (µg/dL), mean (SD)	214.8 (46.6)
TIBC (µg/dL), mean (SD)	271.5 (37.5)
TSAT (%), mean (SD)	21.5 (9.1)
hs-CRP (mg/dL), median (IQR)	0.200 (0.050–0.545)
IL-6 (pg/mL), median (IQR)	7.40 (4.40–10.40)^a^
Serum EPO (mIU/mL), median (IQR)	31.00 (13.10–63.30)^a^

SD, standard deviation; BMI, body mass index; CKD, chronic kidney disease; GNRI, geriatric nutritional risk index; ERI, erythropoiesis-stimulating agent resistance index; IQR, interquartile range; RBC, red blood cell; MCV, mean corpuscular volume; MCH, mean corpuscular hemoglobin; MCHC, mean corpuscular hemoglobin concentration; AST, aspartate aminotransferase; ALT, alanine aminotransferase; γ-GTP, γ-glutamyl transpeptidase; UIBC unsaturated iron-binding capacity; TIBC, total iron-binding capacity; TSAT, transferrin saturation; hs-CRP, high sensitive C-reactive protein; IL-6, interleukin-6; EPO, erythropoietin

^a^*n* = 58 for calculation due to missing values

### Correlation between serum hepcidin-25 levels and ERIs

**Fig. 1:**
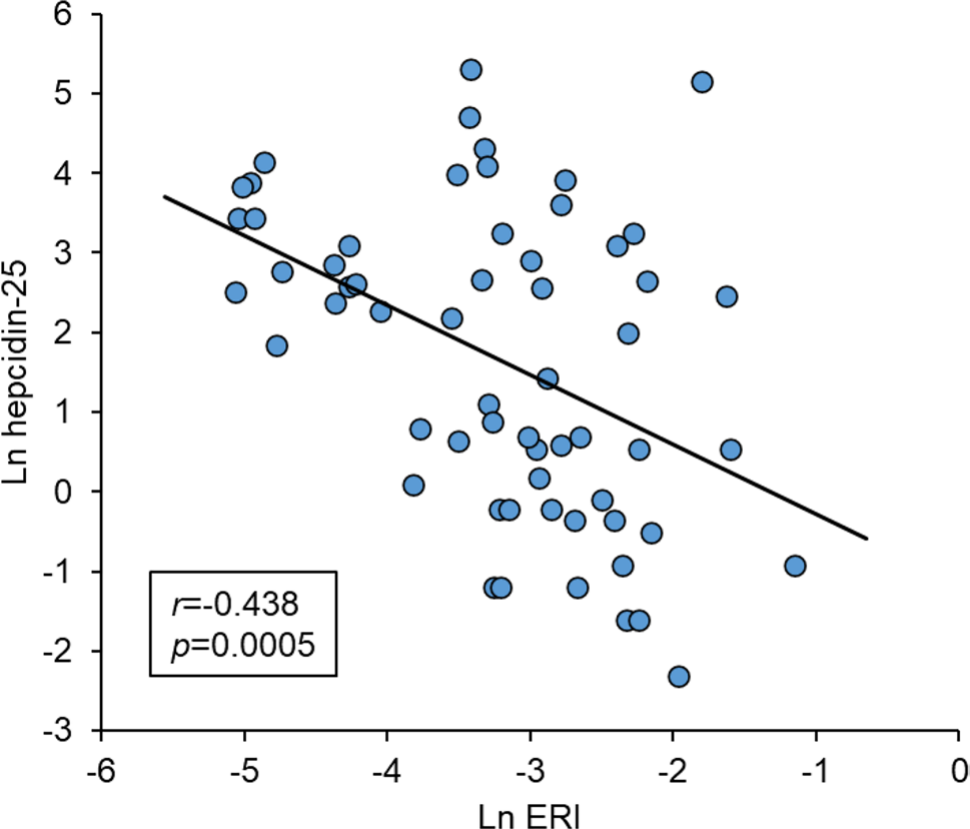
Relationship between hepcidin-25 and ERI. ERI, erythropoiesis-stimulating agent resistance index; Ln, natural logarithm

There was a significant negative correlation coefficient between natural logarithm (Ln) hepcidin-25 levels and Ln ERIs in Japanese patients receiving HD (–0.438, *p* = 0.0005) (Fig. 1 and Table 2).

**Table 2:** Correlations between hepcidin-25 levels and patient background including ESA dose, dialysis condition and laboratory measurement parameters

Parameter	Ln Hepcidin-25	
	*r*	*p* value
**Patient background**		
Ln ERI	− 0.438	0.0005
Ln ESA dose	− 0.408	0.0012
Age	− 0.250	0.0537
BMI	0.276	0.0328
GNRI	0.053	0.6885
**Dialysis condition**		
Ln history of hemodialysis	0.067	0.6129
Kt/V	− 0.265	0.0407
Dialysis time	− 0.202	0.1209
**Laboratory measurement**		
Hemoglobin	− 0.202	0.1224
Hematocrit	− 0.380	0.0028
RBC	− 0.386	0.0023
MCV	0.219	0.0921
MCH	0.335	0.0089
MCHC	0.415	0.0010
Platelet	0.233	0.0726
Ln total bilirubin	0.035	0.7930
Ln AST	− 0.192	0.1426
Ln ALT	− 0.159	0.2256
Ln γ-GTP	− 0.091	0.4882
Cholinesterase	0.293	0.0233
Serum iron	0.390	0.0021
UIBC	− 0.563	< 0.0001
TIBC	− 0.470	0.0002
TSAT	0.493	< 0.0001
Ln ferritin	0.623	< 0.0001
Ln hs-CRP	0.302	0.0188
Ln IL-6	0.073	0.5872
Ln serum EPO	− 0.453	0.0004

Ln, natural logarithm; ERI, erythropoiesis-stimulating agent resistance index; ESA, erythropoiesis-stimulating agent; BMI, body mass index; GNRI, geriatric nutritional risk index; RBC, red blood cell; MCV, mean corpuscular volume; MCH, mean corpuscular hemoglobin; MCHC, mean corpuscular hemoglobin concentration; AST, aspartate aminotransferase; ALT, alanine aminotransferase; γ-GTP, γ-glutamyl transpeptidase; UIBC, unsaturated iron-binding capacity; TIBC, total iron-binding capacity; TSAT, transferrin saturation; hs-CRP, high sensitive C-reactive protein; IL-6, interleukin-6; EPO, erythropoietin

### Relationships between serum hepcidin-25 levels and patient background parameters, dialysis condition parameters, laboratory measurement parameters, and iron repletion categories

**Fig. 2:**
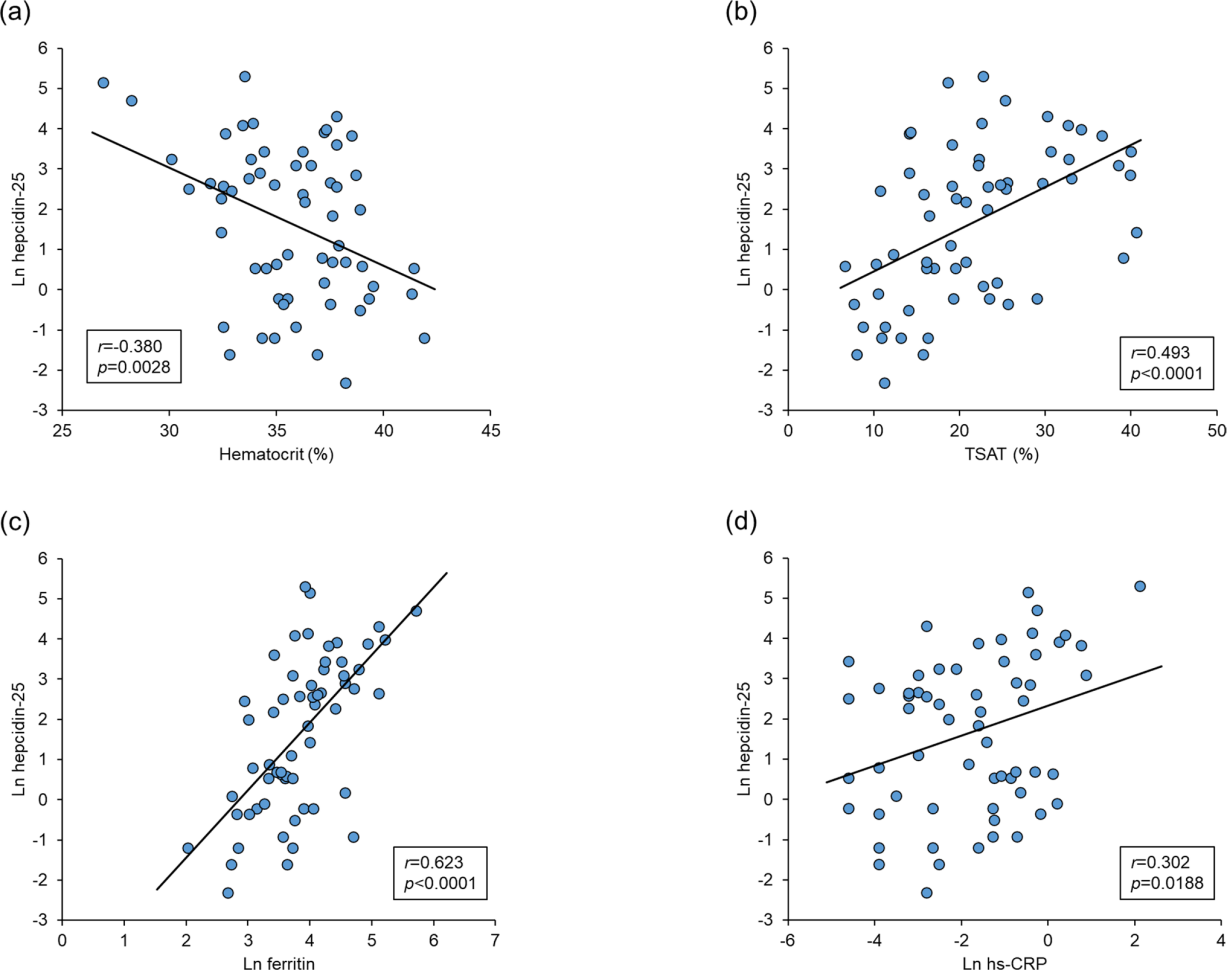
Relationships of hepcidin-25 with **a** hematocrit, **b** TSAT, **c** ferritin, and **d** hs-CRP. The solid line represents the regression line. TSAT, transferrin saturation; hs-CRP, high sensitive C-reactive protein; Ln, natural logarithm

Ln hepcidin-25 levels were negatively correlated with Ln ESA dose, Kt/V, hematocrit, red blood cells, unsaturated iron-binding capacity, total iron-binding capacity, and Ln serum EPO levels (Table 2 and Fig. 2a) and were positively correlated with body mass index, mean corpuscular hemoglobin, mean corpuscular hemoglobin concentration, cholinesterase, serum iron, TSAT, Ln ferritin, and Ln hs-CRP (Table 2 and Fig. 2b-d).

Hepcidin-25 levels were significantly higher in patients who received oral iron-containing preparations than in those not receiving these preparations (Table 3). In terms of iron repletion categories, hepcidin-25 levels were significantly higher in patients in Group I (ferritin ≥ 100 ng/mL and TSAT ≥ 20%) and Group II (ferritin < 100 ng/mL and TSAT ≥ 20%) compared with Group IV (ferritin < 100 ng/mL and TSAT < 20%); however, the numbers of cases were imbalanced among the categories. There was no significant difference in hepcidin-25 levels within categories regarding sex, primary disease of CKD, diabetes, heart failure, or use of intravenous iron.

**Table 3:** Serum hepcidin-25 levels according to patient background parameters and iron repletion categories

Parameter	Category	*n*	Median (IQR), ng/mL	*p* value^*^
Sex	Male	41	8.90 (1.70–22.00)	0.3777
	Female	19	2.40 (0.70–25.70)	
Primary disease of CKD	CGN	18	11.45 (1.70–22.00)	CGN vs. DN, 0.9517; CGN vs. NC, 0.6577; CGN vs. Others/Unknown, 0.9245; DN vs. NC, 0.9223; DN vs. Others/Unknown, 0.7275; NC vs. Others/Unknown, 0.4999
	DN	22	6.85 (0.80–17.50)	
	NC	10	2.10 (0.60–14.10)	
	Others/Unknown	10	16.80 (1.70–62.50)	
Diabetes mellitus	Present	32	6.85 (0.85–33.90)	0.9468
	Absent	28	9.30 (1.70–62.50)	
Heart failure	Present	3	1.20 (0.20–36.80)	0.4555
	Absent	57	8.90 (1.10–22.00)	
Intravenous iron	user	12	1.90 (0.80–14.50)	0.2518
	non-user	48	10.30 (1.15–28.30)	
Oral iron-containing preparations	user	12	33.85 (11.85–58.10)	0.0064
	non-user	48	2.30 (0.80–16.75)	
Iron repletion	Group I	6	39.75 (16.00–74.30)	Group I vs. Group II, 0.1616; Group I vs. Group III, 0.7494; Group I vs. Group IV, 0.0085; Group II vs. Group III, 0.9916; Group II vs. Group IV, 0.0206; Group III vs. Group IV, 0.9821
	Group II	24	13.35 (2.10–28.30)	
	Group III	2	24.40 (0.40–48.40)	
	Group IV	28	1.75 (0.50–10.20)	

Group I, Ferritin ≥ 100 ng/mL and TSAT ≥ 20%; Group II, Ferritin < 100 ng/mL and TSAT ≥ 20%; Group III, Ferritin ≥ 100 ng/mL and TSAT < 20%; Group IV, Ferritin < 100 ng/mL and TSAT < 20%; IQR, interquartile range; CKD, chronic kidney disease; CGN, chronic glomerulonephritis; DN, diabetic nephropathy; NC, nephrocalcinosis; TSAT, transferrin saturation

^*^Mann–Whitney U test for sex, diabetes mellitus, heart failure, intravenous iron and oral iron-containing preparations. Steel–Dwass test for primary disease of CKD and iron repletion

### Parameters affecting hepcidin-25 levels

We investigated the parameters affecting hepcidin-25 in HD patients by multivariate regression analysis of variables correlated with hepcidin-25 in simple regression analysis. The results of the multiple regression analysis for Ln hepcidin-25 levels are shown in Table 4, with a coefficient of determination of 0.707. Ln ERI, hematocrit, TSAT, Ln ferritin, Ln hs-CRP, and oral iron-containing preparations displayed a dominant relationship with Ln hepcidin-25 levels.

**Table 4:** Multiple regression analysis of parameters affecting hepcidin-25 levels

Independent variable	Coefficient (95% CI)	SE	*p* value
Intercept	2.430 (− 2.055, 6.915)	2.236	0.2820
Ln ERI	− 0.564 (− 0.891, − 0.201)	0.172	0.0025
Hematocrit	− 0.172 (− 0.273, − 0.070)	0.051	0.0013
TSAT	0.044 (0.005, 0.082)	0.019	0.0278
Ln Ferritin	0.839 (0.361, 1.318)	0.239	0.0009
Ln hs-CRP	0.441 (0.252, 0.629)	0.094	<.0001
Oral iron-containing preparations	1.122 (0.386, 1.857)	0.367	0.0035
R^2^ = 0.707			

Ln, natural logarithm; CI, confidence interval; SE, standard error; ERI, erythropoiesis-stimulating agent resistance index; TSAT, transferrin saturation; hs-CRP, high sensitive C-reactive protein

## Discussion

We evaluated the association between serum hepcidin-25 levels and hyporesponsiveness to ESAs in Japanese patients receiving HD, and showed that serum hepcidin-25 levels were significantly negatively correlated with ERI, as an indicator of hyporesponsiveness to ESAs. In addition to the ERI, hematocrit, TSAT, serum ferritin, hs-CRP, and use of oral iron-containing preparations were also associated with serum hepcidin-25 levels.

This study found a negative correlation between serum hepcidin-25 levels and ERIs, consistent with some previous reports [11, 12, 15]. Hepcidin-25 levels are also affected by ESA dose in patients with CKD [16, 17], and stimulation of erythropoiesis by EPO administration suppressed hepcidin mRNA levels in animal experiments [18]. The negative correlation between ERI and hepcidin-25 levels may be due to the downregulation of hepcidin-25 levels by ESAs. One recent study reported that hepcidin-25 levels were lower in ESA-hyporesponsive than in ESA-responsive Japanese HD patients, although multivariate regression analysis showed no significant association [12]. The present study enrolled patients according to ESA dose categories, and the population was considered to be well-balanced in terms of ESA doses, which may have enabled appropriate analysis and contributed to the detection of a significant difference in multivariate regression analysis. This point is considered a strength of current study.

Regarding the parameters associated with hepcidin-25 levels in this study, we detected a significant positive correlation with hs-CRP levels. Hepcidin-25 in the liver is induced by inflammatory cytokines such as IL-6 [19], and levels were previously shown to correlate positively with inflammatory markers such as CRP [10, 20]. Although several reports also found a correlation between hepcidin-25 and IL-6 [15, 21], others, including the current study, found no such association [20, 22, 23]. This apparent discrepancy may be due to differences in the timing of hepcidin-25 and IL-6 measurements. The present study included patients undergoing stable HD treatment and excluded patients with severe comorbidities, suggesting that this was a stable patient population without acute inflammation. Tomosugi et al. reported an association between hepcidin-25 and IL-6 in patients with severe inflammation, but no such association in stable HD patients [23]. As previously reported [10, 20], serum hepcidin-25 levels in the current study were correlated with CRP levels, reflecting inflammatory responses. In patients with inflammation, HIF-PHIs may improve iron utilization and may thus be used to treat anemia through downregulation of hepcidin-25 [24].

In the present analysis, hematocrit, ferritin, and TSAT were independently associated with hepcidin-25 levels, indicating a relationship between red blood cell indices and iron status and hepcidin regulation. Hematocrit has previously been reported to be negatively correlated with hepcidin-25 levels [25], which is consistent with the present results. Regarding the association between hepcidin-25 and erythropoiesis, increased erythropoiesis increases erythroferrone production by erythroblasts, and erythroferrone in turn acts directly on the liver to repress hepcidin-25 [26]. The results of the current study support an association between erythropoiesis and hepcidin-25. In terms of ferritin and TSAT, several clinical studies have shown positive correlations between these parameters and hepcidin [16, 17, 22], and the present study consistently showed positive correlations of hepcidin-25 levels with these parameters. These results are reasonable given the role of hepcidin-25 as a major regulator of systemic iron homeostasis [27]. According to a recent report, it is suggested that hepcidin-25 levels could predict responsiveness to HIF-PHIs in CKD patients while TSAT or ferritin do not [28]. It might be possible to avoid unnecessary medications if appropriate drug was chosen by hepcidin-25 measurement during anemia treatment.

Patients who received oral iron-containing preparations had higher hepcidin-25 levels than those without such preparations, suggesting that these preparations affected hepcidin-25 levels. Oral iron-containing preparations have previously been reported to increase iron-related parameters, such as ferritin, and decreased ferritin levels were associated with decreased hepcidin-25 levels, indicating that serum ferritin is a positive predictor of serum hepcidin-25. In the present study, we only measured serum ferritin levels once in patients who received oral iron-containing preparations, and the changes in serum ferritin were thus unknown. Increased iron availability (from oral iron), however, leads to upregulation of hepcidin-25 [29], and elevated hepcidin-25 levels reduce iron absorption and iron release from stores, possibly contributing to ESA hyporesponsiveness [30]. High hepcidin-25 levels are thought to inhibit iron absorption and to be associated with defective iron utilization [31, 32]. HIF-PHIs have recently become available in Japan for the treatment of anemia in patients with CKD, with a different mechanism of action from ESAs. HIF-PHIs not only induce the production of endogenous EPO but also improve iron utilization through indirect down-regulation of hepcidin-25. The present study showed that the administration of oral iron-containing preparations was associated with high hepcidin-25 levels. Iron absorption may be inhibited in patients with high hepcidin-25 levels, thus reducing the effectiveness of iron supplementation. HIF-PHIs may thus improve iron utilization and thereby improve anemia in patients receiving oral iron-containing preparations, representing an optimal therapeutic option.

The median hepcidin-25 level detected in the current study was 8.15 ng/mL (mean was 21.60 ± 38.10 ng/mL) using the LIA method, which has shown a good correlation with conventional liquid chromatography/tandem mass spectrometry [13]. Hepcidin-25 levels in this study tended to be lower than those recently reported for 268 Japanese HD patients (42.9 ± 38.7 ng/mL) [16]. This may have been affected by the high proportion of patients with HDF. HD generally only removes low-molecular-weight substances effectively, and does not effectively remove middle-molecular-weight substances, while HDF shows improved clearance of middle-molecular-weight substances from the blood, by combining convection clearance by hemofiltration and diffusion clearance by HD. Given that hepcidin-25 is a middle-molecular-weight peptide (25 amino acids), it may be more likely to be removed from the blood in patients receiving HDF than in those receiving HD. A previous report accordingly found that serum hepcidin-25 levels were lower in patients receiving on-line HDF compared with patients receiving HD [33]. The Dialysis Outcomes and Practice Patterns Study (DOPPS) reported that 23% of dialysis patients in Japan were receiving HDF [34], while the percentage in the present study was as high as 93.3%.

This study had some limitations. First, it was a cross-sectional study with a small sample size. Especially for subgroup analyses (e.g., iron repletion categories), statistical power is limited due to the imbalance in group sizes. Second, the patient characteristics were limited because they were derived from a single center. Third, patients with serious comorbidities in the liver or other organs were excluded to ensure patient safety. Finally, potential confounding in unmeasured covariates, such as genetic variation or association with comorbidities, was not considered.

In conclusion, the present analysis showed that hepcidin-25 levels were negatively correlated with ERI among Japanese patients receiving HD. Regarding other factors, hematocrit, TSAT, serum ferritin, hs-CRP levels, and the administration of oral iron-containing preparations might also be associated with serum hepcidin-25 levels. Hepcidin-25 may thus be an important factor in the treatment of anemia in patients with CKD; however, further large-scale and prospective studies are needed to clarify the associations between hepcidin-25 levels and hyporesponsiveness to ESAs and other background factors.

## Data Availability

The data generated and/or analyzed in the present study are available from the corresponding author on reasonable request.
